# Remote Hemorrhage in the Cerebellum and Temporal Lobe after Lumbar Spine Surgery

**DOI:** 10.1155/2015/972798

**Published:** 2015-08-03

**Authors:** Shotaro Watanabe, Seiji Ohtori, Sumihisa Orita, Kazuyo Yamauchi, Yawara Eguchi, Yasuchika Aoki, Junichi Nakamura, Masayuki Miyagi, Miyako Suzuki, Gou Kubota, Kazuhide Inage, Takeshi Sainoh, Jun Sato, Yasuhiro Shiga, Koki Abe, Kazuki Fujimoto, Hiroto Kanamoto, Gen Inoue, Takeo Furuya, Masao Koda, Akihiko Okawa, Kazuhisa Takahashi, Masashi Yamazaki

**Affiliations:** Department of Orthopaedic Surgery, Graduate School of Medicine, Chiba University, 1-8-1 Inohana, Chuo-ku, Chiba 260-8670, Japan

## Abstract

Cerebellar hemorrhage remote from the site of surgery can complicate neurosurgical procedures. However, this complication after lumbar surgery is rare. Furthermore, hemorrhage in both the cerebellum and the temporal lobe after spine surgery is rarer still. Herein we present a case of remote hemorrhage in both the cerebellum and the temporal lobe after lumbar spine surgery. A 79-year-old woman with a Schwannoma at the L4 level presented with low back and bilateral leg pain refractory to conservative management. Surgery was undertaken to remove the Schwannoma and to perform posterior fusion. During the surgery, the dura mater was removed in order to excise the Schwannoma. Reconstruction of the dura mater was performed; postoperatively the patient had a cerebrospinal fluid leak. Five days after surgery, clouding of consciousness started gradually, and hemorrhage in the cerebellum and the temporal lobe was revealed by computed tomography. Emergent evacuation of the hemorrhage was performed and the patient recovered consciousness after the surgery. Leakage of cerebrospinal fluid may have induced this hemorrhage. While rare, intracranial hemorrhage after spine surgery can occur, sometimes requiring emergent intervention.

## 1. Introduction

Brain hemorrhage is a well known complication after surgery, but remote cerebellar hemorrhage (RCH) is rare, particularly as a complication after spinal surgery. The phenomenon was first described by Chadduck after a cervical laminectomy [1]. RCH after spinal surgery has a reported incidence of 0.08%, and intraoperative cerebrospinal fluid (CSF) loss following dural tear may precipitate it [2–5]. Other areas of the brain may similarly be affected after spine surgery, as is reported in the literature. Herein we present a case of remote hemorrhage in both the cerebellum and the temporal lobe after lumbar spine surgery. We review our case as well as the 16 RCH cases after lumbar spine surgery reported in the literature.

## 2. Case Presentation

**Figure 1:**
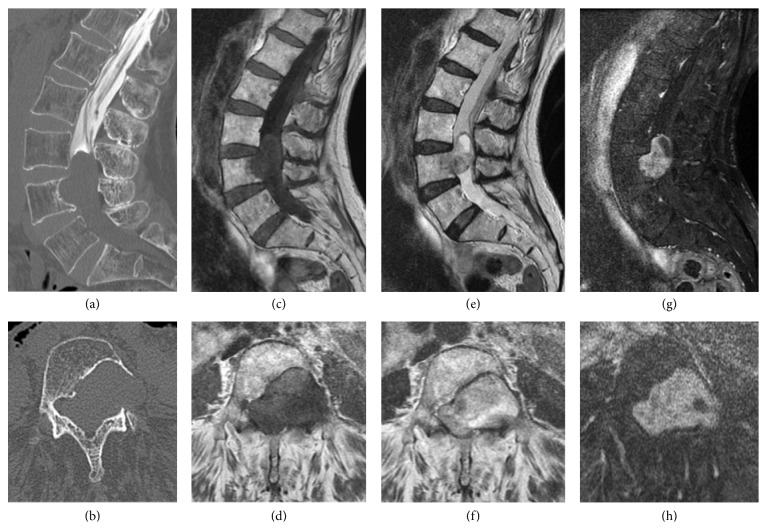
CT after myelography showing bone scalloping of the L4 vertebra body and left pedicle, and central spinal canal stenosis at L3 and L4 ((a) and (b)). MRI on the T1- ((c) and (d)) and T2-weighted images ((e) and (f)) before surgery. T1-weighted MRI scan after gadolinium administration showed enhancement of the spinal tumor ((g) and (h)).

**Figure 2:**
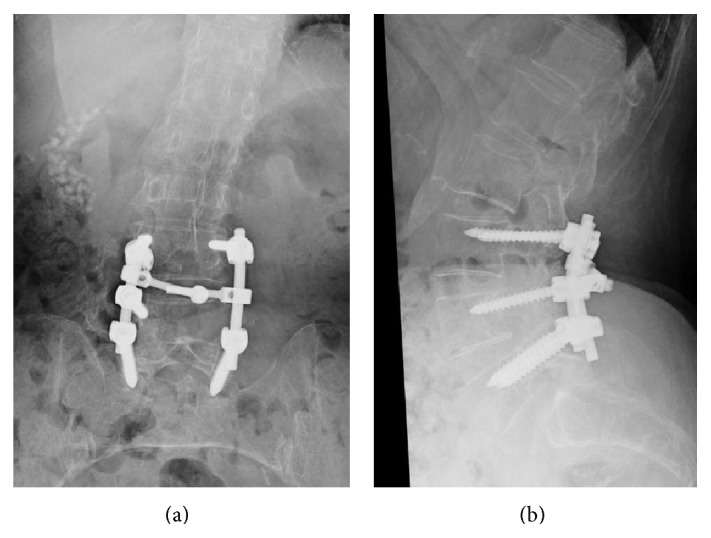
Removal of the tumor and posterolateral fusion was performed. (a) Anteroposterior view, and (b) lateral view.

Informed consent was received from the patient for this report. In June 2010, a 79-year-old woman presented with a 10-year history of low back pain and bilateral leg pain. Visual analogue scale (VAS) of low back pain was 5 (worst 10), right leg pain was 7, and left leg pain was 8. Motor weakness using Manual Muscle Testing (MMT) was not observed, and sensory examination using the pin prick test confirmed an abnormality of bilateral L5 dermatomes. Deep tendon reflexes were normal in both legs. There were no urinary symptoms. Bilateral straight leg raising tests results were negative. Computed tomography (CT) after myelography showed scalloping of the L4 vertebra (Figure 1). Magnetic resonance imaging (MRI) showed a low signal intensity on T1-weighted images and a high signal intensity on T2-weighted images. Gadolinium enhanced MRI showed a high signal intensity on T1-weighted images (Figure 1). Preoperative diagnosis was a Schwannoma. Because conservative treatment was not effective, surgery was planned. Surgery entailed removal of the tumor and posterolateral fusion. As the tumor was quite large (3.5 × 4.0 cm), part of dura mater (2.5 × 3.0 cm) was also removed. The dura mater was reconstructed with a synthetic patch. Posterolateral fusion was performed using pedicle screws and local bone which was grafted between the transverse processes from L3 to L5 (Figure 2). Postoperatively, leakage of CSF was noted.

**Figure 3:**
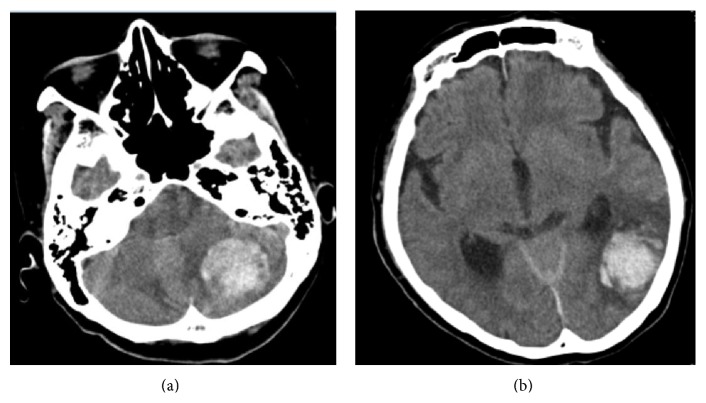
Hemorrhage was observed in both the left cerebellum (a) and the temporal lobe (b) 5 days after spine surgery on CT.

**Figure 4:**
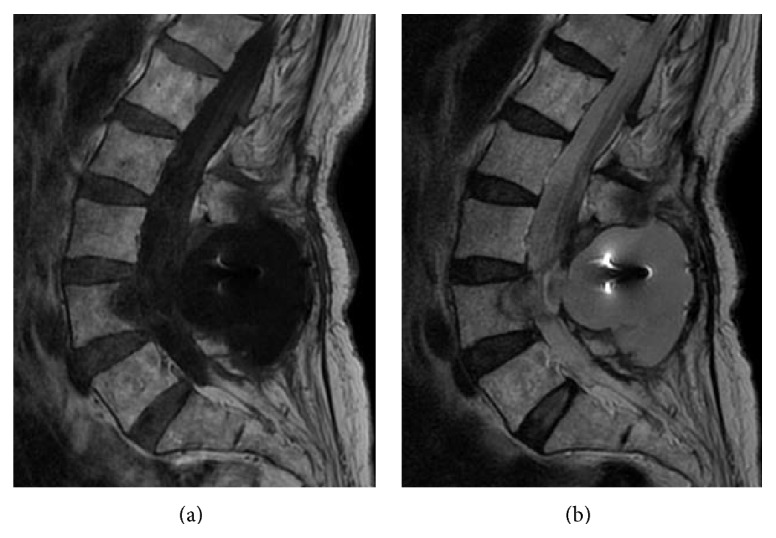
Cerebrospinal fluid accumulation 2 years after surgery. Sagittal images show low intensity on the T1-weighted images (a) and high intensity fluid on the T2-weighted images (b) 2 years after surgery.

Five days postoperatively, clouding of consciousness started gradually. We found headache and gait disturbance however did not find any change of her vital sign. Intracranial hemorrhage was revealed by CT (Figure 3). Hemorrhage was observed in the cerebellum and the temporal lobe. We consulted the neurosurgery service and they performed emergent evacuation of the hematoma. After the procedure, the patient recovered full consciousness but did have some degree of dysphagia. One month after surgery, she could walk with the help of a cane and was discharged. Twenty-four months after surgery, the patient could walk unassisted and no longer had dysphagia nor any other neurologic sequelae. However, MRI showed CSF accumulation within her back (Figure 4).

## 3. Discussion

In the current study, we present a case of remote hemorrhage in both the cerebellum and the temporal lobe after lumbar spine surgery.

There have been 16 RCH cases after lumbar spine surgery reported in the literature. Common features are shown in Table 1 [2, 3, 6–17]. Most patients were elderly. An important common feature among these cases and the current case was damage of the dura mater intraoperatively with subsequent CSF leak (15 of 17 cases). Dural rupture during surgery and CSF hypovolemia are the main risk factors for RCH. In the 2 cases without definite dural damage, the author suggested the possibility that the dura had been damaged [2].

Khalatbari et al. and Hashidate suggest that RCH is probably a manifestation of cerebellar venous hemorrhage and infarction [6, 18]. Cerebellar “sag,” which is a result of excessive CSF leakage, has been proposed to cause stretching and occlusion of the superior vermian veins. In patients with insufficient venous collaterals, this may cause venous infarction, subsequently leading to hemorrhagic transformation [6, 18]. Another explanation is a rise in transmural venous pressure associated with CSF drainage and intracranial hypotension [6, 18].

Hemorrhage in the cerebellum has been the most commonly reported location for a remote bleed after spine surgery; however, other areas have been reported to be involved including the temporal lobe in 2 cases [8, 17], the 4th ventricle in 1 case [6], both the 3rd and the 4th ventricles in 1 case [14], and the subarachnoid space in 1 case [2].

In 17 cases including our case, typical symptoms after RCH included headache, nausea, vomiting, gait disturbance, and depressed consciousness [2, 3, 6–17]. Depressed consciousness was observed in 9 of 17 cases [3, 6, 7, 11, 13, 14, 16] (Table 2). In the present case, she showed headache, gait disturbance, and depressed consciousness. The symptoms of RCH appeared within 48 hours after lumbar spine surgery in 14 cases [3, 6–14, 16, 17] (Table 1).

In 6 of 17 cases, brain surgery including craniotomy for removal of hematoma and insertion of an external ventricular drain (EVD) was needed [7, 11, 13, 14, 16] (Table 2). All of these cases included patients with depressed consciousness (Table 2). Postoperative outcomes included death at 16 days in one patient [6], neurologic deficit in 5 cases [11, 12, 14, 16], complete recovery of symptoms in 10 cases (as in the current case) [2, 3, 6–10, 13, 17], and unknown outcome in 1 case [13]. In the 6 cases in which the symptoms did not completely recover, neurologic impairment was not severe.

## 4. Conclusion

We present a case of remote hemorrhage in both the cerebellum and the temporal lobe after lumbar spine surgery. We conclude that age and damage to the dura matter with CSF leak are risk factors for RCH. Surgical evacuation of hemorrhage may be necessary in patients with severe symptoms or depressed consciousness.

## Figures and Tables

**Table 1:** 

Author	Age, sex	Opened dura	Amount of CSF	With other hemorrhages	Generation time
Khalatbari et al. [6]	53 y, M 75 y, M	○ ○	550 mL 150 mL	— The 4th ventricular	8 hr after op. in op.
Andrews and Koci [7]	36 y, M	○	250–400 mL/day	—	36 hr after op.
Thomas et al. [8]	38 y, F	○	700 mL/day	Temporal lobe	Right after op.
Friedman et al. [9]	56 y, F	○	500 mL/day	—	2 days after op.
Konya et al. [10]	48 y, F	○	580 mL/day	—	12 hr after op.
Cevik et al. [2]	79 y, F 68 y, F	Unknown Unknown	unkown unkown	— Subarachnoid	3 days after op. 7 days after op.
Karaeminogullari et al. [11]	73 y, F	○	A large volume	—	2 days after op.
Cavanilles-Walker et al. [12]	65 y, F	○	A little volume	—	48 hr after op.
Farag et al. [13]	43 y, F	○	A large volume	—	36 hr after op.
Lee et al. [3]	63 y, F	○	300 mL	—	15 min. after op.
Nam et al. [14]	61 y, M	○	Unknown	The 3rd and 4th ventricular	1 day after op.
Çalişaneller et al. [15]	67 y, F	○	Unknown	—	8 days after op.
Gul et al. [16]	64 y, F	○	300 mL/6 h	—	24 hr after op.
Hempelmann and Mater [17]	69 y, F	○	450 mL/day	temporal lobe	1 day after op.

**Table 2:** 

Author	Symptoms	Brain surgery	Postoperative courses
Khalatbari et al. [6]	Headache, vomiting, and low level of consciousness	—	Complete recover
	Low level of consciousness	—	Dead
Andrews and Koci [7]	Low level of consciousness	Done	Complete recover
Thomas et al. [8]	Nausea, headache	—	Complete recover
Friedman et al. [9]	Headache, nausea, and walking disturbance	—	Complete recover
Konya et al. [10]	Headache, nausea	—	Complete recover
Cevik et al. [2]	Headache	—	Complete recover
	Headache, nausea	—	Complete recover
Karaeminogullari et al. [11]	Low level of consciousness	Done	Survival of slight symptoms
Cavanilles-Walker et al. [12]	Walking disturbance	—	Survival of slight symptoms
Farag et al. [13]	Low level of consciousness	Done	Not mentioned
Lee et al. [3]	Headache, low level of consciousness	—	Complete recover
Nam et al. [14]	Headache, nausea, and low level of consciousness	Done	Survival of slight symptoms
Çalişaneller et al. [15]	Headache, walking disturbance	—	Complete recover
Gul et al. [16]	Low level of consciousness	Done	Survival of slight symptoms
Hempelmann and Mater [17]	Headache, nausea	—	Complete recover
